# Deep learning-based differentiation of non-tuberculous mycobacterial lung disease and pulmonary tuberculosis using chest CT

**DOI:** 10.3389/fmed.2026.1786346

**Published:** 2026-04-21

**Authors:** Bingchuan Hu, Bin Wu, Yuwei Zhou, Zherui Shao, Qingning Wang, Binyu Luo, Zhuo Yu, Dawei Zheng

**Affiliations:** 1 Department of Thoracic Surgery, The Affiliated Lihuili Hospital of Ningbo University, Ningbo, China; 2 College of Medical Science, Ningbo University, Ningbo, China; 3 Hangzhou Fuyangyan Medical Imaging Technical Service Studio, Hangzhou, China

**Keywords:** artificial intelligence, chest CT, deep learning, non-tuberculous mycobacterial, tuberculosis

## Abstract

**Background:**

Differentiating non-tuberculous mycobacterial lung disease (NTM-LD) from pulmonary tuberculosis (PTB) remains a significant clinical challenge owing to their overlapping clinical and imaging features, despite markedly different therapeutic strategies.

**Methods:**

A total of 409 patients with microbiologically confirmed diagnoses were retrospectively enrolled and randomly divided into a training set (*n* = 329; NTM-LD: 171, PTB: 158) and an independent test set (*n* = 80; NTM-LD: 41, PTB: 39). After lung segmentation with nnU-Net, images were intensity-normalized and resampled to 256 × 256 × 128 voxels. A 3D ResNeXt-based classifier was developed and compared against six mainstream deep learning architectures: ResNet, SENet, DenseNet, ShuffleNet, Transformer, and Swin Transformer. Model performance was evaluated using the area under the receiver operating characteristic curve (AUC), accuracy, sensitivity, specificity, and F1 score.

**Results:**

The proposed 3D ResNeXt model achieved the highest performance, with an AUC of 0.89 and accuracy of 0.89 on the training set, and an AUC of 0.83 and accuracy of 0.84 on the independent test set. DeLong’s test confirmed statistically significant superiority over all six comparator architectures on the test set (all *p* < 0.05). Gradient-weighted Class Activation Mapping (Grad-CAM) visualizations highlighted disease-specific features, including nodular bronchiectasis and tree-in-bud opacities in NTM-LD, as well as thick-walled cavitary lesions in PTB.

**Conclusion:**

The 3D ResNeXt model demonstrated superior and interpretable performance in differentiating NTM-LD from PTB on chest CT. It holds promise as a valuable clinical decision-support tool, although prospective multicenter validation is warranted.

## Introduction

Nontuberculous mycobacterial lung disease (NTM-LD) and pulmonary tuberculosis (PTB) are both significant chronic respiratory infectious diseases worldwide. They frequently exhibit substantial overlap in clinical symptoms and imaging manifestations, such as cough, sputum production, fatigue, and radiographic findings including nodules, cavities, and bronchiectasis (1, 2). This similarity poses a major challenge for accurate clinical differential diagnosis. However, the etiology, treatment strategies, and management approaches for the two diseases differ fundamentally. PTB is caused by *Mycobacterium tuberculosis*, is contagious, and requires standardized anti-tuberculosis chemotherapy. In contrast, NTM-LD is primarily caused by environmental nontuberculous mycobacteria, is typically non-contagious, and necessitates long-term, multi-drug regimens tailored to the specific species, most of which exhibit intrinsic resistance to conventional anti-tuberculosis drugs (3–5). Therefore, early and accurate differentiation is crucial to avoid misdiagnosis, optimize clinical decision-making, and improve patient outcomes.

Currently, the gold standard for differential diagnosis relies on pathogenic microbial culture and molecular testing. However, these methods are time-consuming, often requiring several weeks, and have limited sensitivity, which fails to meet the clinical demand for rapid diagnosis (6). Chest computed tomography (CT), a core imaging modality for evaluating pulmonary diseases, provides high-resolution morphological details. Studies suggest that NTM-LD and PTB may show subtle differences in lesion distribution patterns and morphological features on CT images. For example, NTM-LD more frequently involves the right middle lobe and lingula with diffuse bronchiectasis, whereas cavities in PTB are more commonly located in the apical and posterior segments of the upper lobes (7, 8). Nevertheless, identification of these features remains highly subjective and heavily dependent on radiologist experience, resulting in diagnostic inconsistency and limited efficiency.

In recent years, deep learning (DL) technology, particularly convolutional neural networks (CNNs), has advanced significantly in medical image analysis. DL models can automatically learn and extract high-level features from large volumes of imaging data that often surpass human visual perception. They have demonstrated performance comparable to, or even exceeding that of, experienced radiologists in various chest CT tasks, such as pulmonary nodule classification and pneumonia detection (9–13). This progress offers a promising technological pathway for developing objective, quantitative, and efficient auxiliary tools to differentiate NTM-LD from PTB. Although radiomics approaches have been explored in this domain (14–16), research using end-to-end deep learning frameworks that directly differentiate the two diseases from raw CT images remains in its early stages, and its full potential has yet to be realized.

To this end, the present study aimed to develop and validate a deep learning model based on chest CT for the automatic differentiation of NTM-LD and PTB. We retrospectively collected chest CT imaging data from patients with microbiologically confirmed NTM-LD and PTB, constructed a deep convolutional neural network model, and comprehensively evaluated its diagnostic performance, robustness, and clinical utility through internal validation.

## Materials and methods

### Patients

**Figure 1:**
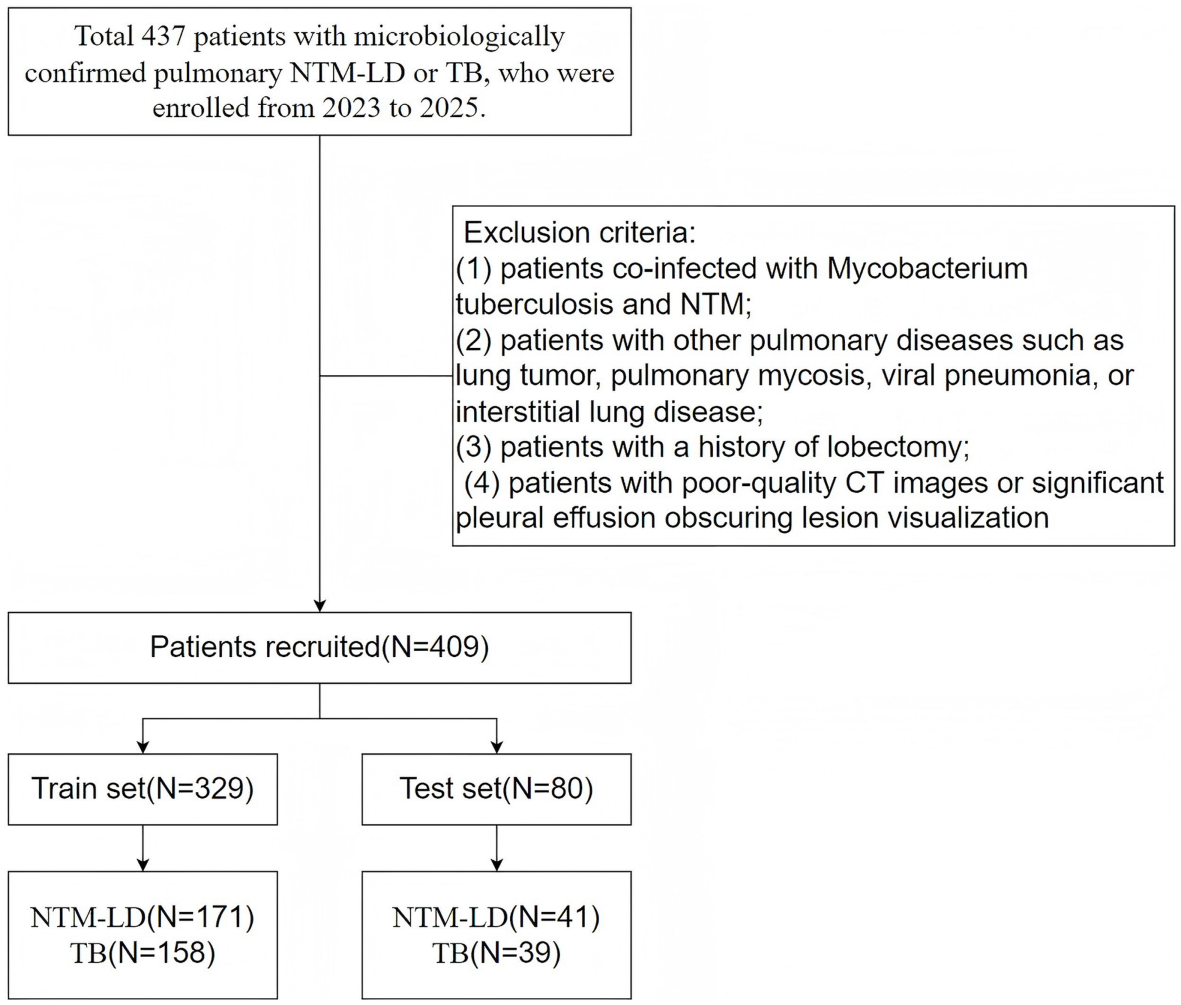
The process of inclusion and exclusion of study subjects.

This retrospective study included 437 patients with microbiologically confirmed pulmonary NTM-LD or PTB who were enrolled from The Affiliated Lihuili Hospital of Ningbo University between 2023 and 2025. Exclusion criteria were as follows: (1) co-infection with *Mycobacterium tuberculosis* and NTM; (2) concurrent other pulmonary diseases such as lung tumor, pulmonary mycosis, viral pneumonia, or interstitial lung disease; (3) history of lobectomy; and (4) poor-quality CT images or significant pleural effusion obscuring lesion visualization. Figure 1 presents the flowchart outlining the inclusion and exclusion process, which ultimately yielded a final cohort of 409 patients. The dataset was randomly divided into a training cohort (*n* = 329; NTM-LD: 171, PTB: 158) and a test cohort (*n* = 80; NTM-LD: 41, PTB: 39) at an 8:2 ratio using the random package. Pathological examination served as the reference standard. The study protocol was approved by the Hospital Medical Ethics Committee.

### Image acquisition and preprocessing

CT scans were performed on multi-detector CT scanners with the following parameters: tube voltage 120 kVp, tube current 100–200 mAs, pitch 1.0–1.2, and collimation 0.625–1 mm. Images were reconstructed with a lung kernel at a slice thickness of 1 mm.

**Figure 2:**
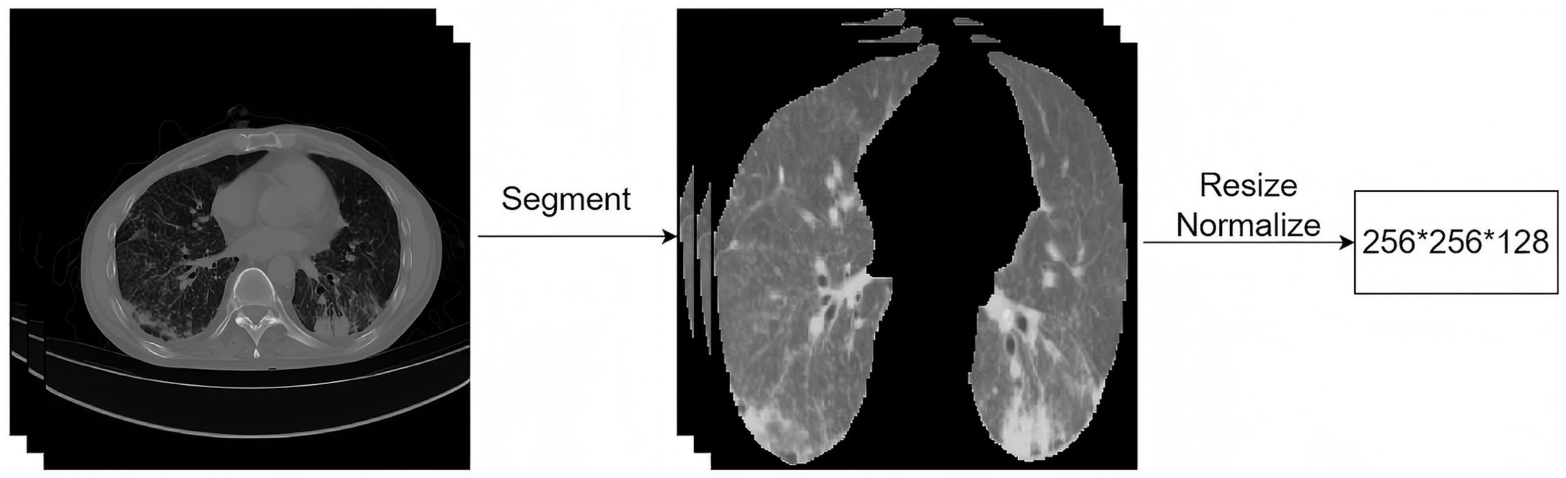
Image preprocessing.

To reduce the impact of varying image quality on model performance, standardized image preprocessing was conducted. As shown in Figure 2, lung fields were first segmented using nnU-Net (17), which was trained on COVID-19 and chest CT datasets (https://www.kaggle.com/datasets/hgunraj/covidxct/data, Dice = 0.98). The images were then resampled to 1.0 mm isotropic spacing, rescaled to 256 × 256 × 128 voxels, intensity-clipped to the range of [−1,000, 400] Hounsfield Units (HU), and normalized. Data augmentation was applied online to the training set only using the MONAI package. The applied techniques included flipping, rotation (±10°), addition of Gaussian noise, and elastic deformation.

### Model architecture

**Figure 3:**
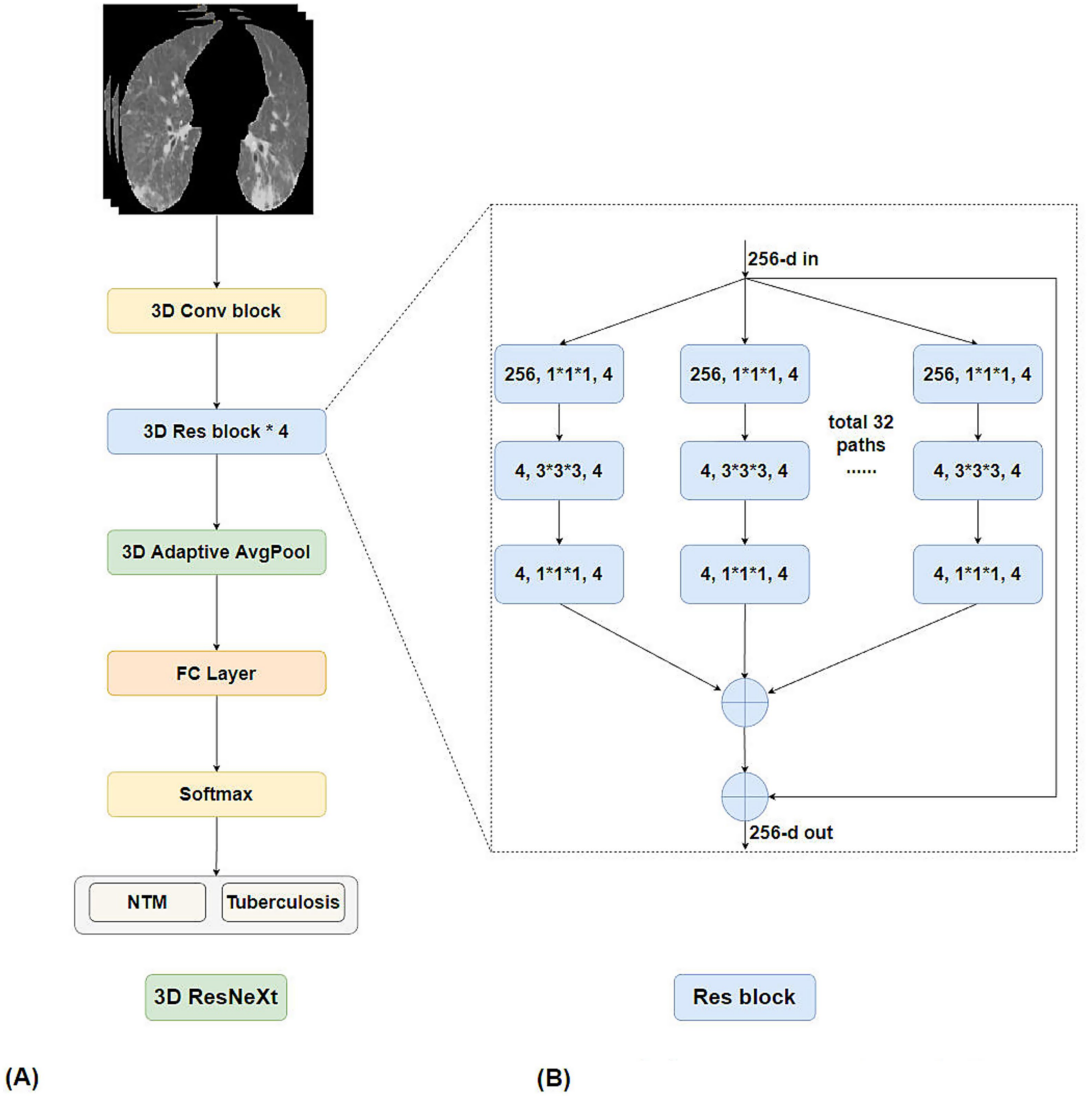
3D ResNeXt network structure diagram. **(A)** Main framework of 3D ResNeXt. **(B)** Detailed components of Res block.

For the classification task, the 3D ResNeXt architecture (18) was employed. The core of 3D ResNeXt builds upon the residual network framework and incorporates grouped convolutions to enhance efficiency. Its fundamental building block is a residual module optimized for high “cardinality”. This module divides the input feature maps into multiple parallel low-dimensional groups. Within each group, 3D convolution, batch normalization, and ReLU activation are performed independently. The outputs from all groups are then concatenated and merged. This design significantly strengthens the model’s capacity to learn multi-branch feature representations without a substantial increase in parameters (Figure 3).

The overall network is constructed by stacking multiple such modules into a deep architecture. Within each module, a skip connection directly adds the input to the output, effectively mitigating the vanishing gradient problem in deep networks. The network begins with a basic 3D convolutional layer and a down-sampling layer for initial feature extraction. This is followed by several stages of residual modules. Each stage may contain multiple identical modules, and spatial down-sampling is performed between stages to expand the receptive field. The network terminates with a global average pooling layer and a fully connected layer for classification output. This structure enables 3D ResNeXt to efficiently capture multi-level spatial contextual information from 3D medical images. The final layer was configured for binary classification (NTM-LD vs. PTB). Dropout (rate = 0.5) and batch normalization were applied throughout the network to regularize and stabilize training.

### Model evaluation

A comparative analysis was conducted utilizing ResNet (19), SENet (20), DenseNet (21), ShuffleNet (22), Transformer (23), and Swin-Transformer (24) models. Model performance was quantitatively assessed using standard metrics: accuracy, precision, recall, F1-score, and AUC. For qualitative visual analysis, receiver operating characteristic (ROC) curves were compared, and confusion matrices were generated to delineate classification details.


$$\text{Accuracy}=\frac{\mathrm{TN}+\mathrm{TP}}{\mathrm{TP}+\mathrm{TN}+\mathrm{FP}+\mathrm{FN}}$$

$$\text{Precision}=\frac{\mathrm{TP}}{\mathrm{TP}+\mathrm{FP}}$$

$$\text{Recall}=\frac{\mathrm{TP}}{\mathrm{TP}+\mathrm{FN}}$$

$$F1=\frac{2^{*}{\text{Precision}}^{*}\text{Recall}}{\text{Precision}+\text{Recall}}$$

*TP* and *FN* represent the number of NTM patients diagnosed as NTM and pulmonary tuberculosis, respectively; *TN* and *FP* represent the number of pulmonary tuberculosis patients diagnosed as pulmonary tuberculosis and NTM, respectively.

### Statistical analysis

All calculations and statistical analyses were conducted in a Linux environment (Ubuntu 20.04) using the following hardware configuration: an Intel 4215FR CPU clocked at 3.20 GHz, 64 GB DDR4 memory, and an RTX 4060 Ti graphics card. The programming language utilized was Python 3.6.13 from the Python Software Foundation. We employed the PyTorch deep learning framework along with key packages such as torch (version 1.10.1), torchvision (version 0.11.2), and scikit-learn (version 0.20.4).

## Results

### Patients

A total of 409 valid imaging datasets were ultimately included for analysis, comprising 197 cases from the pulmonary tuberculosis (PTB) group and 212 cases from the NTM-LD group. The mean age was 56.3 ± 17.4 years, with 197 male and 212 female participants. Detailed demographic and clinical characteristics are presented in Table 1.

**Table 1:** The distribution of cases on the training and validation sets.

Clinical features	Train set (*n* = 329)	Test set (*n* = 80)	*p*-value
Age (years), (mean + SD)	63.6 ± 16.2	61.8 ± 15.5	0.441
Sex, *n* (%)			0.589
Male	159 (48.33)	38 (47.50)	
Female	170 (51.67)	42 (52.50)	
Disease classification, *n* (%)			0.634
NTM-LD	171 (51.98)	41 (51.25)	
TB	158 (48.02)	39 (48.75)	

### Model development and hyperparameter optimization

The optimal hyperparameters for the 3D ResNeXt architecture were determined through a systematic grid search and ablation analysis. We evaluated various combinations of batch size, learning rate, and optimizer configurations to maximize the Area Under the Curve (AUC) on the validation set. After rigorous experimentation, the final configuration was established as follows: batch size = 8, learning rate = 0.002 (decayed by a factor of 0.8 every 20 epochs), Adam optimizer, cross-entropy loss, with a maximum of 500 epochs and early stopping implemented with a patience of 30 epochs.

### Model performance evaluation

The proposed 3D ResNeXt model was compared with six mainstream deep learning architectures (ResNet, SENet, DenseNet, ShuffleNet, Transformer, and Swin-Transformer) on the same training set (*n* = 329) and an independent test set (*n* = 80). As shown in Table 2, the 3D ResNeXt model achieved the best or highly competitive results across all evaluation metrics.

**Table 2:** Performance comparison of different deep learning models on the training and test sets.

Models	Train set (*n* = 329)					Test set (*n* = 80)				
	AUC	ACC	SEN	SPE	F1	AUC	ACC	SEN	SPE	F1
ResNet	0.84	0.83	0.85	0.83	0.84	0.82	0.76	0.75	0.80	0.78
SENet	0.82	0.84	0.85	0.84	0.84	0.81	0.78	0.78	0.78	0.78
DenseNet	0.83	0.84	0.84	0.86	0.85	0.73	0.76	0.76	0.78	0.77
ShuffleNet	0.84	0.83	0.85	0.82	0.83	0.78	0.79	0.79	0.80	0.80
Transformer	0.86	0.84	0.85	0.85	0.85	0.82	0.79	0.80	0.78	0.79
Swin-transformer	0.83	0.85	0.86	0.85	0.86	0.77	0.80	0.82	0.78	0.80
3D ResNeXt (Ours)	0.89	0.89	0.91	0.88	0.89	0.83	0.84	0.83	0.85	0.84

ACC, accuracy; SEN, sensitivity; SPE, specificity.

On the training set, the 3D ResNeXt demonstrated excellent learning capability, achieving an AUC of 0.89, accuracy of 0.89, sensitivity of 0.91, specificity of 0.88, and F1 score of 0.89. More importantly, on the independent test set, the model maintained the strongest generalization performance and robustness, with an AUC of 0.83 and accuracy of 0.84. In contrast, the other models exhibited more pronounced performance degradation when transitioning from the training set to the test set; for example, the AUC of DenseNet decreased from 0.83 to 0.73. This result indicates that the 3D ResNeXt architecture adopted in this study can more effectively learn discriminative and less overfitting-prone feature representations from 3D CT images.

To rigorously compare model performance, DeLong’s test was performed on the AUCs derived from the independent test set. The proposed 3D ResNeXt model demonstrated statistically significant superiority over all six comparator architectures (all *p* < 0.05; Table 3).

**Table 3:** DeLong’s test for statistical significance of AUC differences among models on the test set.

Models	ResNet	SENet	DenseNet	ShuffleNet	Transformer	Swin-transformer	3D ResNeXt (Ours)
ResNet		0.0435	0.6194	0.0478	0.2277	0.0155	0.0175
SENet	0.0435		0.4746	0.2901	0.1702	0.023	0.0428
DenseNet	0.6194	0.4746		0.6803	0.0291	0.0967	0.0332
ShuffleNet	0.0478	0.2901	0.6803		0.1947	0.0469	0.0273
Transformer	0.2277	0.1702	0.0291	0.1947		0.6986	0.0079
Swin-transformer	0.0155	0.023	0.0967	0.0469	0.6986		0.0079
3D ResNeXt (Ours)	0.0175	0.0428	0.0332	0.0273	0.0079	0.0079	

### Confusion matrix and ROC curve analysis

**Figure 4:**
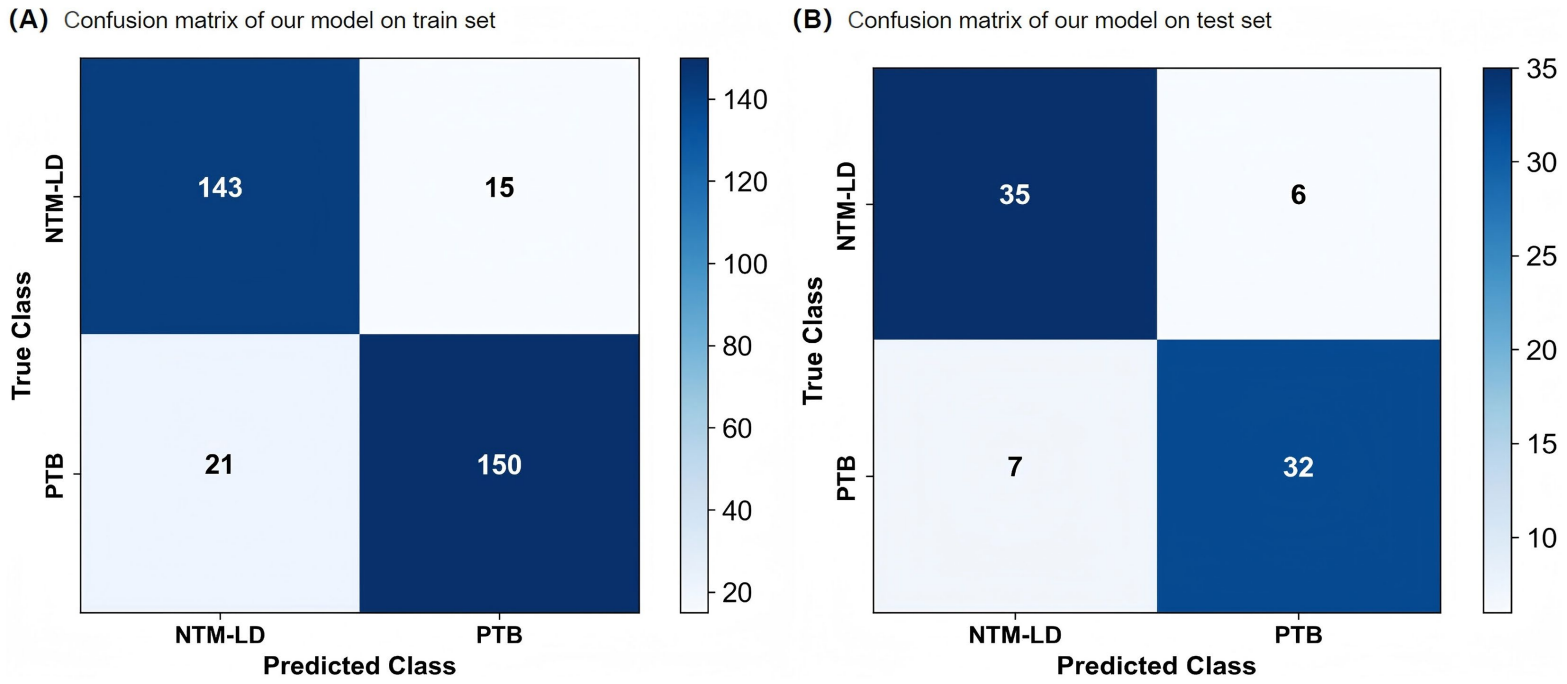
Confusion matrix of our model. **(A)** Train set. **(B)** Test set.

**Figure 5:**
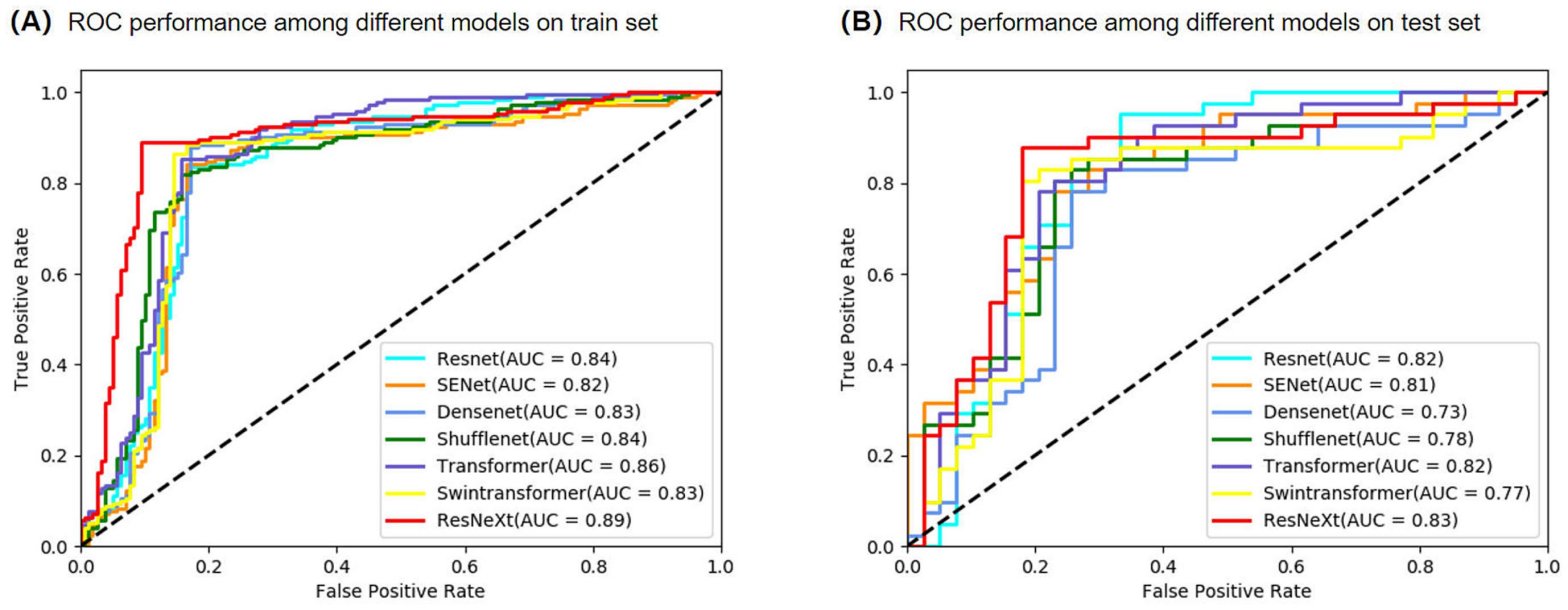
Comparison of ROC performance among different models. **(A)** Train set. **(B)** Test set.

To further evaluate classification details, confusion matrices (Figure 4) and receiver operating characteristic (ROC) curves (Figure 5) were generated for both the training and test sets. On the test set, the model misclassified only 13 out of 80 cases: 6 NTM-LD cases were misclassified as PTB, and 7 PTB cases were misclassified as NTM-LD. The similar number of errors for both classes indicates that the model did not exhibit significant class bias and maintained relatively balanced classification decisions. Furthermore, the area under the ROC curve (AUC = 0.83) confirmed the model’s strong overall discriminative ability.

### Grad-CAM visualization and interpretability

**Figure 6:**
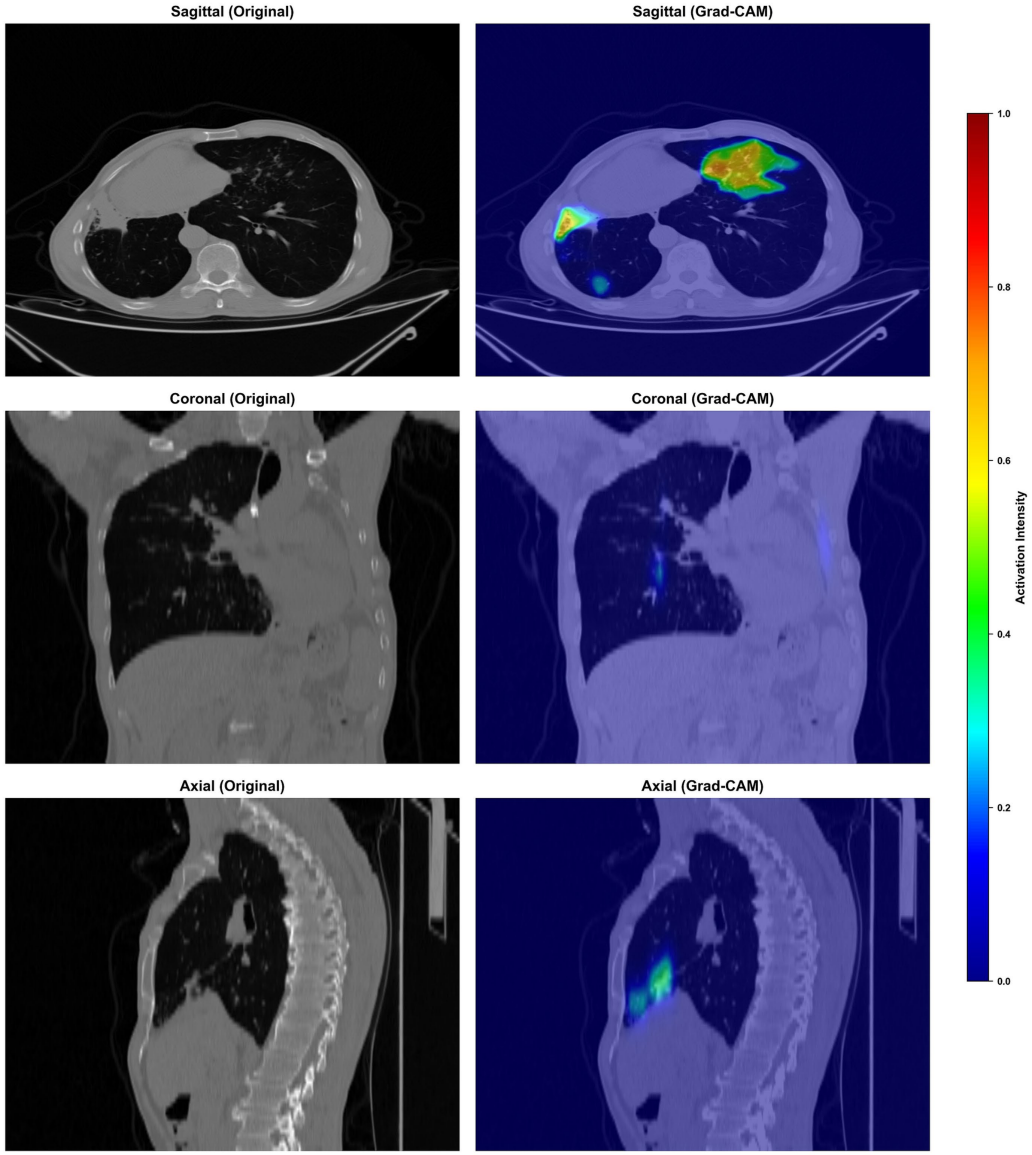
Grad-CAM heatmaps overlaid on sagittal, coronal, and axial chest CT views for NTM-LD case.

**Figure 7:**
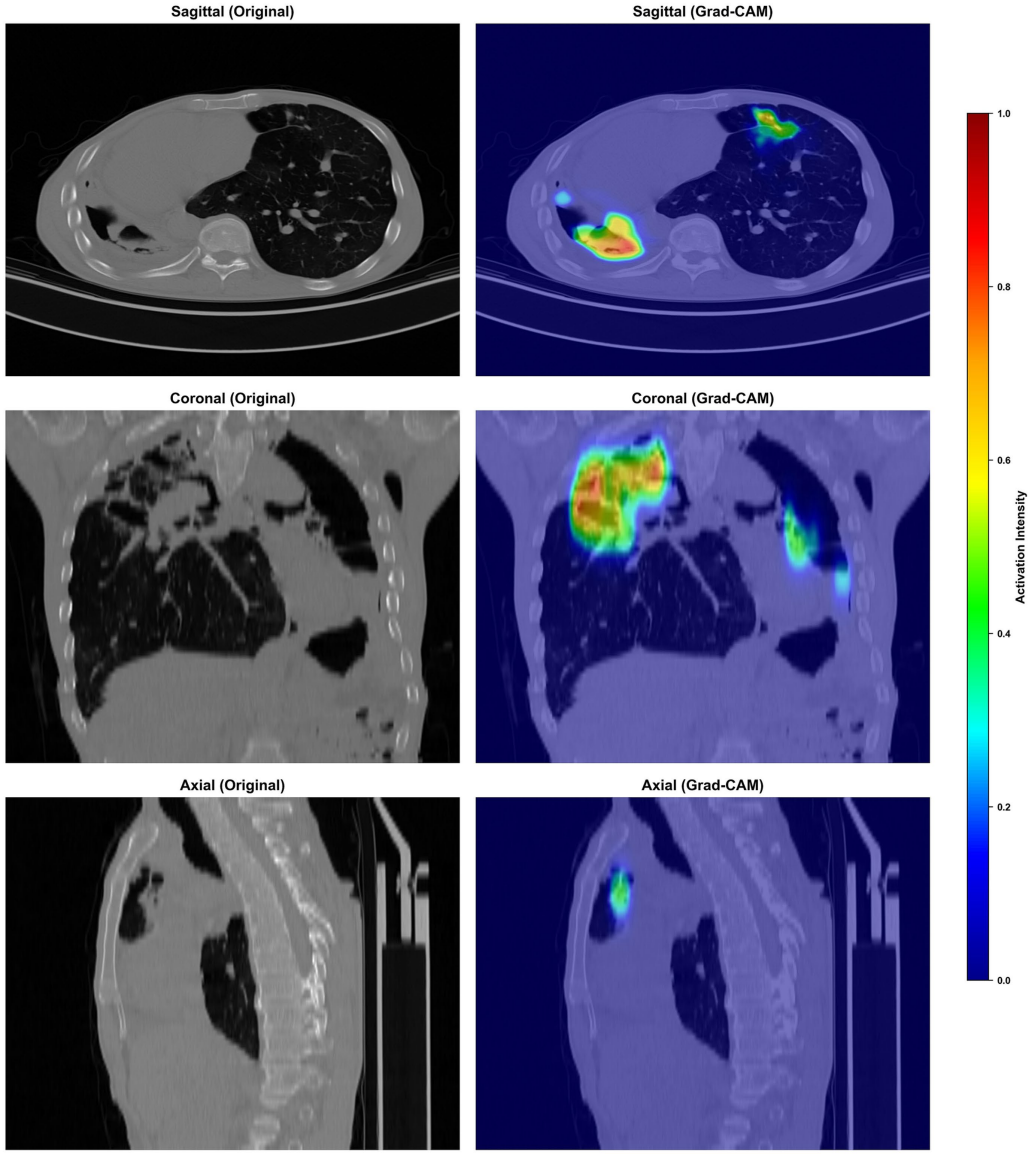
Grad-CAM heatmaps overlaid on sagittal, coronal, and axial chest CT views for PTB cases.

To enhance model transparency and clinical interpretability, Gradient-weighted Class Activation Mapping (Grad-CAM) was applied. Figures 6, 7 present representative Grad-CAM heatmaps overlaid on sagittal, coronal, and axial chest CT views for NTM-LD and c cases, respectively.

In NTM-LD cases, the model primarily attended to regions of nodular bronchiectasis, tree-in-bud opacities, and centrilobular nodules. In PTB cases, high activation was observed predominantly in thick-walled cavitary lesions and consolidative opacities, most commonly located in the upper lobes. These attention patterns are highly consistent with established radiological hallmarks of each disease. From an explainable artificial intelligence (XAI) perspective, the Grad-CAM visualizations provide visual evidence that the network learns clinically meaningful features rather than spurious correlations, thereby increasing radiologist confidence and facilitating integration into clinical diagnostic workflows.

### Ablation experiments

To validate the contribution of key components in our methodology, a series of ablation experiments were conducted (Table 4).

**Table 4:** Performance of the 3D ResNeXt model with different ablated preprocessing strategies on the test set.

Models	Test set (*n* = 80)				
	AUC	ACC	SEN	SPE	F1
Removing lung region segmentation	0.83	0.84	0.83	0.85	0.84
Reducing image resolution	0.81	0.81	0.81	0.83	0.82
Pneumonia pre-segmentation	0.81	0.82	0.82	0.83	0.82
3D ResNeXt (Ours)	0.79	0.79	0.8	0.81	0.80

ACC, accuracy; SEN, sensitivity; SPE, specificity.

#### Effect of lung segmentation

When precise lung field segmentation (nnU-Net) was removed and the model was trained directly on raw thoracic CT images, the test accuracy decreased by 3.2%. This indicates that eliminating irrelevant background interference (e.g., soft tissue, bones) is crucial for improving the model’s focus on pulmonary lesions.

#### Effect of image resolution

Reducing the input voxel resolution from 256 × 256 × 128 resulted in a 2.7% decrease in test accuracy. This highlights the importance of high-resolution 3D data for capturing subtle lesion morphology, such as fine bronchiectasis or small cavities.

#### Effect of pre-segmentation strategy

Furthermore, replacing the lung segmentation with a general pneumonia pre-segmentation model^1^ led to a significant 6.3% drop in test accuracy. This underscores that disease-specific preprocessing strategies, such as isolating the entire lung field, are superior to generic lesion-focused segmentation for differentiating complex mycobacterial diseases.

## Discussion

Accurate differentiation between NTM-LD and PTB remains a persistent clinical conundrum due to the substantial overlap in their radiographic phenotypes, despite the requirement for distinct therapeutic pathways (25). Current diagnostic paradigms rely heavily on microbiological culture, a methodological bottleneck that often entails a waiting period of several weeks, potentially delaying the initiation of appropriate treatment (26, 27). Although chest CT provides high-resolution morphological data and specific imaging patterns—such as the predominance of bronchiectasis in NTM-LD versus upper-lobe cavitation in PTB—are recognized, the interpretation of these features remains subjective and highly dependent on the observer’s expertise, leading to inter-observer variability and suboptimal diagnostic efficiency (28, 29). Consequently, there is a compelling clinical imperative for objective, automated decision-support tools to facilitate timely and accurate discrimination.

The advent of deep learning (DL), particularly convolutional neural networks (CNNs), has revolutionized the landscape of medical image analysis by enabling the automated extraction of complex, high-dimensional features that often elude human perception (30–32). Previous attempts to distinguish NTM-LD from PTB have predominantly leveraged radiomics approaches. However, these methods typically require labor-intensive manual region-of-interest (ROI) segmentation and explicit feature engineering, processes that are susceptible to operator-induced variability (33, 34). In contrast, end-to-end deep learning models autonomously learn discriminative features directly from raw image data, offering a more streamlined and potentially robust alternative. While recent studies have explored the feasibility of 2D CNNs for this task (35), the application of 3D architectures for analyzing volumetric CT data remains underexplored. Given the inherently spatial nature of pulmonary pathologies, 3D architectures hold significant potential for capturing crucial contextual information.

In this study, we developed and rigorously validated a 3D ResNeXt model for the binary classification of NTM-LD and PTB. Our model demonstrated superior performance compared to several state-of-the-art 3D architectures, achieving an AUC of 0.83 on the independent test set. The performance superiority of 3D ResNeXt can be attributed to its cardinality-optimized architecture. By employing grouped convolutions within residual modules, the model efficiently learns a diverse repertoire of feature representations across multiple parallel pathways. This architectural advantage enhances its capacity to discern subtle and complex parenchymal patterns—specifically, the tree-in-bud opacities and multifaceted bronchiectasis characteristic of NTM-LD versus the consolidative and cavitary changes typical of PTB—without a prohibitive increase in computational complexity. Furthermore, our ablation studies empirically validated the necessity of our preprocessing pipeline, highlighting that precise lung segmentation and data augmentation are pivotal for achieving robust generalization.

Interpretability is a critical determinant for the clinical adoption of AI tools. The Grad-CAM visualizations generated by our model provide plausible, visually interpretable explanations for its predictions, with high activation maps aligning precisely with established radiological hallmarks. For instance, the model attended to areas of nodular bronchiectasis in NTM-LD and thick-walled cavitary lesions in PTB. This congruence between model attention and clinically relevant imaging features fosters trust and facilitates seamless integration into the radiologist’s workflow, effectively mitigating the “black-box” skepticism often associated with deep learning (36, 37).

### Several limitations in this study warrant consideration

First, the retrospective, single-center design inherently limits the generalizability of our findings. Although we utilized a rigorous internal validation split, the model’s performance must be further validated in prospective, multi-center cohorts encompassing diverse CT protocols and heterogeneous patient demographics to ensure robustness across different clinical settings. Second, the lack of an external validation dataset is a notable constraint. This was primarily due to stringent patient data privacy regulations, which restricted access to external cohorts. Additionally, there is a paucity of publicly available datasets containing CT scans specifically annotated for microbiologically confirmed NTM-LD and PTB, precluding external benchmarking. Third, our study focused on a binary classification task. In real-world clinical practice, the differential diagnosis is broader, necessitating differentiation from other granulomatous diseases, pneumonia, fungal infections, and lung cancer. Future work should expand this framework to multi-class classification. Fourth, while deep learning automates feature extraction, the training process remains contingent on high-quality, meticulously labeled data. Finally, the direct impact of this AI tool on clinical decision-making and patient outcomes requires evaluation through randomized controlled trials comparing AI-assisted diagnosis against standard care.

### Future research should prioritize

(1) External validation across diverse geographic and healthcare settings to assess model portability; (2) Development of integrated diagnostic systems that fuse imaging data with clinical and laboratory variables (radiomics + clinicalomics); (3) Exploration of self-supervised or transfer learning techniques to reduce reliance on large annotated datasets; and (4) Investigation into the model’s utility for predicting disease progression or treatment response.

## Conclusion

The 3D ResNeXt-based DL model achieved robust and interpretable classification of NTM-LD vs. TB on chest CT, outperforming several state-of-the-art models. This approach shows promise as a clinical decision-support tool but requires further validation.

## Data Availability

The raw data supporting the conclusions of this article will be made available by the authors, without undue reservation.

## Footnotes

1
https://github.com/HiLab-git/COPLE-Net
